# Induced Short-Term Hearing Loss due to Stimulation of Age-Related Factors by Intermittent Hypoxia, High-Fat Diet, and Galactose Injection

**DOI:** 10.3390/ijms21197068

**Published:** 2020-09-25

**Authors:** Dong Jun Park, Sunmok Ha, Jin Sil Choi, Su Hoon Lee, Jeong-Eun Park, Young Joon Seo

**Affiliations:** 1 Department of Otorhinolaryngology, Yonsei University Wonju College of Medicine, 20 Ilsan-ro, Wonju, Gangwon-do 26426, Korea; papapdj@gmail.com (D.J.P.); true_choi@yonsei.ac.kr (J.S.C.); tngns6049@daum.net (S.H.L.); 2 Department of Biomedical Laboratory Science, College of Health Sciences, Yonsei University, wonju 26493, Korea; sunmok159@naver.com; 3 Department of Otorhinolaryngology Head and Neck Surgery, Hallym University College of Medicine, Dongtan Sacred Heart Hospital, Hwaseong 18450, Korea; omicsomics@naver.com

**Keywords:** mitochondria dysfunction, reactive oxygen species, hypoxic, d-galactose, high-fat diet, aging, hearing loss

## Abstract

Age-related hearing loss (ARHL) is the most common sensory disorder among the elderly, associated with aging and auditory hair cell death due to oxidative-stress-induced mitochondrial dysfunction. Although transgenic mice and long-term aging induction cultures have been used to study ARHL, there are currently no ARHL animal models that can be stimulated by intermittent environmental changes. In this study, an ARHL animal model was established by inducing continuous oxidative stress to promote short-term aging of cells, determined on the basis of expression of hearing-loss-induced phenotypes and aging-related factors. The incidence of hearing loss was significantly higher in dual- and triple-exposure conditions than in intermittent hypoxic conditions, high-fat diet (HFD), or d-galactose injection alone. Continuous oxidative stress and HFD accelerated cellular aging. An increase in *Ucp2,* usually expressed during mitochondrial dysfunction, was observed. Expression of *Cdh23*, *Slc26a4*, *Kcnq4*, *Myo7a*, and *Myo6*, which are ARHL-related factors, were modified by oxidative stress in the cells of the hearing organ. We found that intermittent hypoxia, HFD, and galactose injection accelerated cellular aging in the short term. Thus, we anticipate that the development of this hearing loss animal model, which reflects the effects of intermittent environmental changes, will benefit future research on ARHL.

## 1. Introduction

Age-related hearing loss (ARHL), also known as presbycusis, is an emerging complication in the aging population worldwide. A gradual decrease of hearing function with increasing age is often perceived as an inevitable part of the human aging process. The overall contribution of ARHL to hearing impairment and decreased quality of life is underestimated. Since the average life expectancy of the population is increasing, hearing loss has significant implications on general health and quality of life [1,2]. Various clinical reports have investigated ARHL. According to the 2015 National Health and Nutrition Examination Survey conducted in the USA, 15% of individuals aged between 40 and 49 years were bilaterally deaf, while 19% of those aged between 50–69 and 43.2% of those aged over 70 years had the same condition [3].

Most studies on ARHL are aimed at its prevention and treatment and require long periods of aging for preclinical evaluation [1]. Factors and causes of ageing have been studied using genetically engineered mice, which have proven useful to uncover the mechanisms of aging and help in the discovery of therapeutic drugs [4]. It has been confirmed that oxidative stress caused by reactive oxygen species (ROS) is involved in cochlear cell death in transgenic mice, with an inhibited expression of the apoptosis-related *bax* gene [4]. In fact, mitochondrial DNA damage caused by aging was observed to induce cell death in the hearing organ. [4]. Similarly, increased expression of superoxide dismutase 1 (*Sod1*) and Cadherin 23 (*Cdh23*) have been reported to be associated with aging [5,6]. It has been shown that mouse strains susceptible to early-onset ARHL carry a specific mutation in the *Cdh23* gene, which encodes a component of the hair cell stereocilia tip-link associated with the mechanoelectrical transduction channels. [5,6]. As for *Sod1*, it has been reported that as the amount of oxidative stress in the cells of the auditory organ increases, the amount of *Sod1* also increases [7,8]. In addition, numerous reports have suggested that in these models, abnormal potassium channels resulting from mass transfer errors cause cell death [9,10]. However, these animal models have certain limitations since they do not reflect the lifestyle of the animals [4,11]. Cumulative damage caused by the surrounding environment contributes to ARHL. Oxidative stress accelerates the aging of auditory cells, ultimately causing hearing loss [12]. Therefore, in order to study the short-term effect of pharmaceutical targets, it is essential to develop a preclinical animal model in which oxidative stress can be induced by environmental changes [13].

We have previously conducted studies related to hearing loss caused by hypoxia. Moreover, an association between decreased oxygen saturation and hearing loss has been reported in the literature [14,15]. Therefore, in this study, we used a method based on the induction model of obstructive sleep apnoea syndrome (OSAS), wherein cell aging is promoted by temporarily blocking the supply of oxygen [16]. This model was designed so that the increase in ROS in the blood rapidly damages the auditory organs. We hypothesized that ARHL would be detected, among other aging phenotype changes, after intermittent exposure to a hypoxic environment.

Mitochondrial damage has been considered responsible for the death of auditory hair cells due to aging [6,17]. Therefore, based on relevant factors identified in the literature, we designed an aging animal model resulting from the exposure to a combination of three different lifestyles [9,18]. It has been reported that mitochondrial dysfunction can be induced by diet modification [19]. A high-fat diet (HFD) results in increased intracellular lipid content, diabetes-induced symptoms, and impairment of mitochondrial function due to oxidative stress. Caloric regulation associated with deafness has been reported to suppress cell aging through the inhibition of *Foxo3* and Sirt1 expression, as well as through the activity of apoptosis-related proteins [20]. In addition, clinical studies have reported that over 50% of diabetes patients suffer from hearing loss and that if diabetes persists for more than 5 years, the hearing loss rate doubles [21]. d-galactose (d-gal) injection animal models, established by administering successive subcutaneous D-gal injections to animals for approximately 6 to 8 weeks, have been frequently used in aging studies. In a study by Guo et al., an increase in aging factors was observed in rodents administered D-gal [22]. In addition, accelerated aging of the brain, kidney, liver, and blood cells has been proven in animal models using the galactose injection technique [23,24,25].

Therefore, we developed an ARHL animal model by changing various aspects of the lifestyle of mice, exposing them to intermittent oxidative stress for a short period of time in order to induce the death of auditory hair cells in the organ of Corti (OC) by stimulating the expression of aging-related factors. This study provides a realistic animal model that can be used to accelerate the development of therapeutic strategies for ARHL in the future.

## 2. Results

### 2.1. Phenotypic Analysis of the Different Groups for Ageing

**Figure 1:**
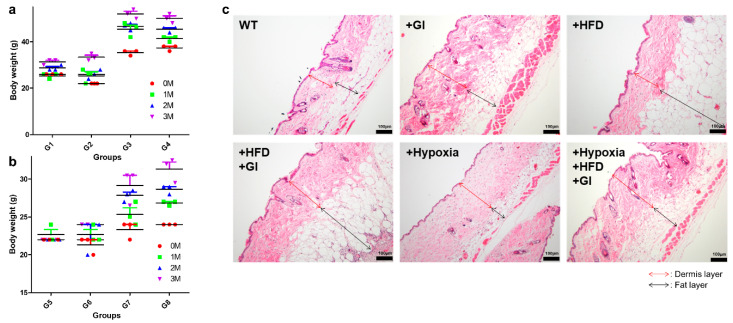
Phenotypic analysis of aging mice under oxidative conditions. (**a**) Measured body weight of the normoxic groups. (**b**) Measured body weight of the hypoxic groups. HFD increased body weight regardless of the hypoxic environment. (**c**) The thickness of the dermis skin layer and the thickness of the fat layer were different. Interestingly, the fat layer was significantly thickened in the HFD groups, but it did not increase in size under hypoxic conditions, although the body weight increased. Rather, the dermis layer became thicker and a lot of wrinkles were observed on the skin surface. Abbreviations: HFD, high-fat diet; WT, wild-type.

The expected phenotype and hearing loss were observed at approximately 12 weeks. The mice were compared according to the three oxidative stress conditions: HFD, galactose injection (GI), and hypoxia. The exposure to the conditions was combinatory, which lead to the formation of 8 groups through a combination of three oxidative stresses. The physical characteristics of the mice in each group were also recorded. In the group under any of the oxidative stress conditions, it was confirmed that the shine of the hair had disappeared, and it became slightly grey in color (Figure S1). The body weight of mice was significantly increased in the normoxic (Figure 1a) and hypoxic HFD groups (Figure 1b) compared to the control group at 3 months. In terms of the different feeding conditions, the control group showed a minor change in body weight of less than 3 g, but the body weight in the HFD group increased by approximately 20 g after 3 months from baseline (0 months; Figure 1a). Under hypoxia, the control group showed a change in body weight of 4 g, and the HFD group showed an increase of over 20 g at 3 months from baseline (Figure 1b). In the group injected with galactose, a change of 8 to 10 g in body weight under normoxic conditions was observed; however, under hypoxic conditions, a change of 3 to 6 g in body weight was observed. Since we cannot determine aging based on changes in body weight alone, skin tissue from mice in each group was obtained to observe the changes in skin phenotype with aging. Interestingly, following an increase in body weight due to dietary conditions, a change in skin thickness was also observed (Figure 1c).

The thicknesses of the dermis skin layer and of the fat layer were measured microscopically, and HFD was confirmed to produce the most notable effect on the fat layer [26,27,28]. The fat layer of mice in the HFD groups was the thickest (Table S1). In addition, hypoxia was confirmed to be the condition that affected the dermis the most. An increase in thickness of about 100 μm was observed under hypoxic conditions, and many deep wrinkles were observed on the skin surface in the GI group.

### 2.2. Oxidative Stress in Serum

**Figure 2:**
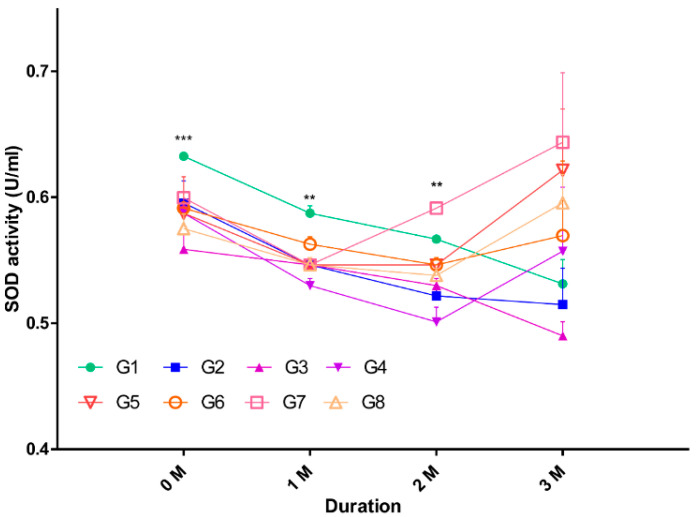
Monthly analysis of SOD activity in the serum from all groups. Serum was collected from mice in each group, and the amount of SOD in serum was measured. Significant values are shown for each month, with ** *p* < 0.005, *** *p* < 0.0005; Abbreviation: SOD, superoxide dismutase.

Oxidative stress has been demonstrated to be the most important factor in causing aging [14,15]. Therefore, an increase in the levels of ROS and superoxide dismutase (SOD) in serum could be a major indicator of aging induction. In this study, we collected serum from mice in each of the groups throughout each month. SOD activity was measured to elucidate the amount of oxidative stress in each group (Figure 2). Interestingly, oxidative stress increased in the hypoxic group at 2 months from baseline. The normoxic group was associated with a tendency of decreased oxidative stress for 3 months (Table 1).

First, the hypoxic control returned a SOD value of 0.6215 ± 0.048, while the SOD value for the normoxic control was 0.5311 ± 0.019. The SOD values for the GI in hypoxic and normoxic groups were 0.5695 ± 0.059 and 0.5148 ± 0.028, respectively. The SOD values for the hypoxic and normoxic groups under HFD were 0.6435 ± 0.055 and 0.4901 ± 0.011, respectively. Finally, when the hypoxic and normoxic groups were exposed to both HFD and GI, the SOD values were 0.5955 ± 0.022 and 0.5572 ± 0.050, respectively. A 0.04 difference in SOD value is a significant error value and can be regarded as a positive trend. In other words, it was confirmed that the amount of SOD in the body increased with longer exposure to oxidative stress, and the difference in SOD values between hypoxic and normoxic groups was between 0.05 and 0.1534. Thus, we confirmed that hypoxia is the main factor for overall SOD activity increase, based on the significant increase in SOD values in G7, G5, and G8 after 3 months.

### 2.3. Comparison of the Hearing Threshold

**Figure 3:**
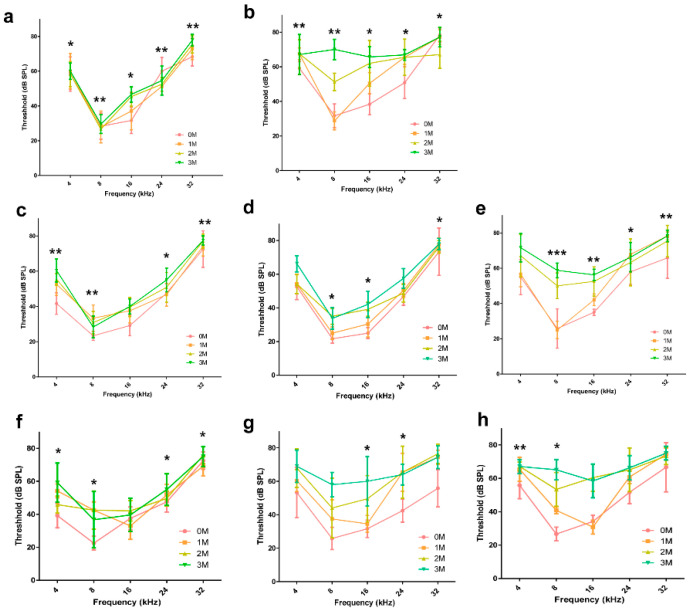
Comparison of the hearing threshold among all groups at various frequencies for each period using the auditory brainstem response (ABR) test. The hearing threshold was found to be greatly decreased under the hypoxic condition. Galactose did not affect hearing loss alone but led to hearing loss in combination with HFD and hypoxic conditions. (**a**) Control group (G1), (**b**) Hypoxia, HFD, GI group (G8), (**c**) GI group (G2), (**d**) HFD group (G3), (**e**) Hypoxia group (G5), (**f**) HFD, GI group (G4), (**g**) Hypoxia, GI group (G6), and (**h**) Hypoxia, HFD group (G7). Significant values are shown, with * *p* < 0.05, ** *p* < 0.005, *** *p* < 0.0005. Abbreviations: HFD, high-fat diet; GI, galactose injection.

Hearing loss due to aging occurs from the highest to the lowest frequencies [4,29]. Hearing thresholds in the mice groups were measured in the frequency range of 4 to 32 kHz by tone-burst auditory brainstem response (ABR; Figure 3). In the control group, the alteration of the hearing threshold was minimal during the 3 months (Figure 3a). We evaluated hearing loss in mice under all three conditions (hypoxia, HFD, and GI) and observed that there was a significant effect on the hearing threshold and that the value of the hearing threshold significantly decreased after 3 months from 35 to 67 dB at 8 kHz and from 31 to 70 dB at 16 kHz (Figure 3b). In the GI- and HFD-only groups, there was no change in the hearing threshold at any frequency (Figure 3c,d). Conversely, there was a significant elevation in the hearing threshold from 25 to 58 dB at 8 kHz and from 35 to 56 dB at 16 kHz in the hypoxic group (Figure 3e). Finally, the last three groups, characterized by exposure to two conditions each, HFD and GI (Figure 3f), hypoxia and GI (Figure 3g), and hypoxia and HFD (Figure 3h), confirmed the dual-exposure effect. The hearing threshold was significantly increased, from 34 ± 3.76 to 67 ± 10.13 dB at 8 kHz and from 38 ± 4.08 to 60 ± 7.33 dB at 16 kHz, in the hypoxic condition. The *p*-value was analyzed by a two-way analysis of variance (ANOVA), and it addressed the significance of hearing loss from 0 to 3 M.

**Figure 4:**
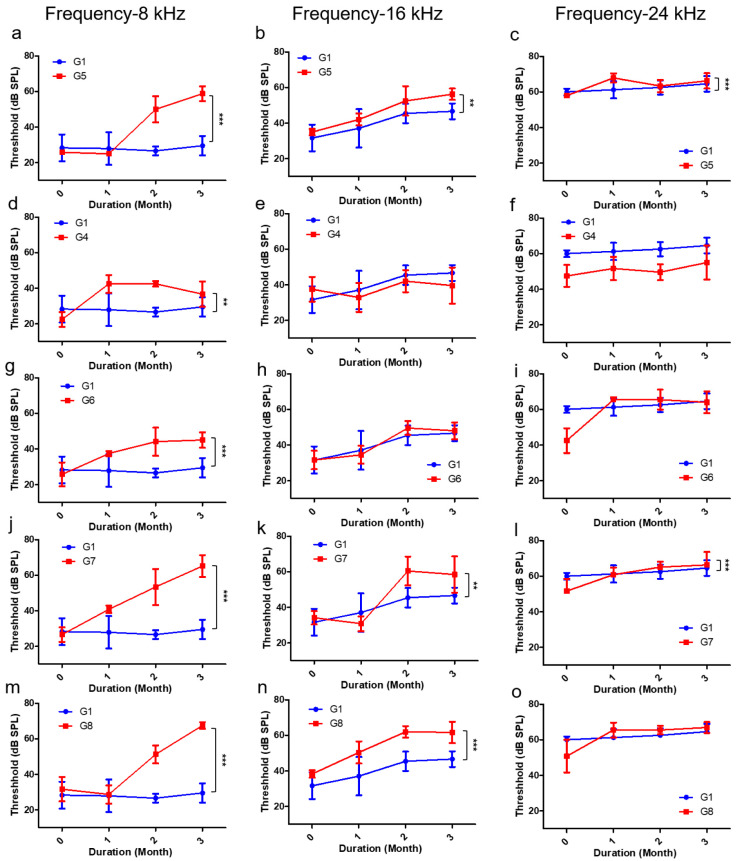
Detailed monthly comparison of hearing thresholds between all the groups and controls for three different frequencies (8, 16, and 24 kHz) by using two-way ANOVA. The analysis of 8 kHz is shown in (**a**,**d**,**g**,**j**,**m**); that of 16 kHz is shown in (**b**,**e**,**h**,**k**,**n**); that of 24 kHz is shown in (**c**,**f**,**I**,**l**,**o**). (**a–c**) G1 and G5, (**d–f**) G1 and G4, (**g–i**) G1 and G6, (**j–l**) G1 and G7, and (**m–o**) G1 and G8. ** *p* < 0.005, *** *p* < 0.0001. Abbreviation: ANOVA, analysis of variance.

The hearing threshold of mice in each group was compared and analyzed among the three conditions through a two-way ANOVA. The analysis was performed by selecting the three frequencies that changed the most: 8, 16, and 24 kHz. The hearing threshold values of the mice in groups G2, G3, and G5 were compared with the control group to analyze the effect of the independent conditions. The results of G2 and G3 showed that the hearing threshold did not decrease significantly compared to that of the control group (Figure S2). In contrast, in the case of G5, hearing loss due to hypoxia showed a significant tendency to appear from the second month onwards (Figure 4a–c). In the group G4, which was the dual-condition group of HFD and GI, hearing did not decrease at any of the frequencies (Figure 4d–f). Interestingly, in the dual-condition groups in which one of the conditions was hypoxia, G6 (plus GI; Figure 4g–i) and G7 (plus HFD; Figure 4j–l), the hearing threshold was significantly reduced from the first month onwards, from about 30 to 60 dB. The triple-condition group, which was characterized by HFD with GI in a low-oxygen environment (G8), showed a tendency of significant decrease in the hearing threshold over a short period of time (Figure 4m–o). Therefore, the hypoxic condition was the one that exerted the maximum effect on hearing loss, while HFD and GI had the least effect.

**Figure 5:**
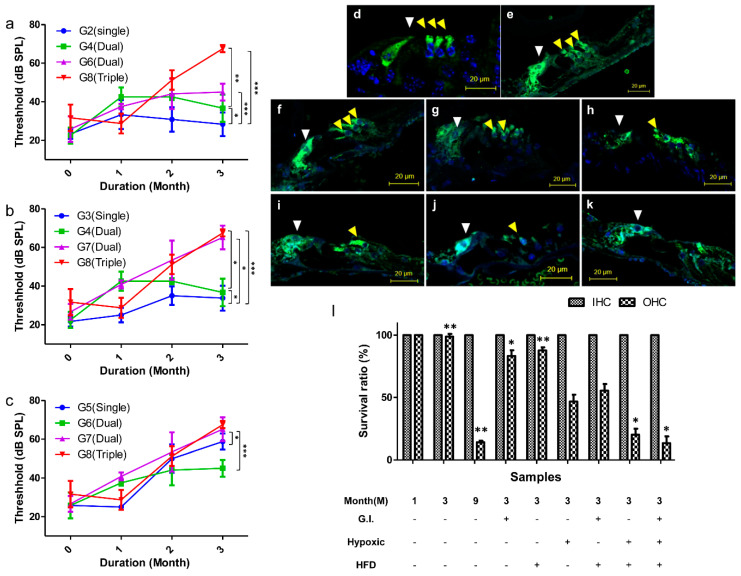
The threshold comparison of single, dual, and triple exposure to three factors (hypoxic condition, HFD, and GI) by two-way ANOVA. (**a**) Hearing threshold analysis of GI between single, double, and triple conditions for 3 months. (**b**) Hearing threshold analysis of HFD between single, double, and triple conditions for 3 months. (**c**) Hearing threshold analysis of the hypoxic condition between single, double, and triple exposure for 3 months. Observation of the damaged OHC and IHC exposed to various conditions. The yellow arrow indicates OHC, and the white arrow indicates IHC in all images (**d–k**). Microscope magnification ×20, scale bar = 20 μM. (**d**) Young mouse, 4 weeks, (**e**) 3 months, (**f**) 3 months + GI, (**g**) 3 months + HFD, (**h**) 3 months + hypoxia, (**i**) 3 months + HFD, GI, (**j**) 3 months + hypoxia, HFD, (**k**) 3 months + hypoxia, HFD, GI, and (**l**) the survival ratio of OHC and IHC in the different groups. Significant values are shown for each month, with * *p* < 0.05, ** *p* < 0.005, *** *p* < 0.0001. Abbreviations: HFD, high-fat diet; GI, galactose injection; ANOVA, analysis of variance; OHC, outer hair cells; IHC, inner hair cells.

For a more specific comparison of the groups, we selected the 8 kHz frequency to assess the determining factors in single-, dual-, and triple-condition exposures. When the results for GI were analyzed, no effective decrease in the hearing threshold was observed, even after including HFD and hypoxia as second exposure conditions (Figure 5a). Analyzing the results of HFD confirmed that hearing loss did not occur with HFD alone, but the hearing threshold did decrease within 2 months when hypoxic conditions were added as a second effect (Figure 5b). In addition, when analyzing the results of hypoxic conditions, it was confirmed that the hearing threshold decreased after 2 months if the hypoxic condition was included (Figure 5c). Therefore, based on the results, it was confirmed that the hypoxic condition and HFD have the greatest effect on hearing loss.

### 2.4. Histological Observations of Hair Cells

We next evaluated the survival rates of auditory hair cells under the different conditions by histological analysis. The survival rate was evaluated by analyzing a major protein, *Myo7a*, present in the auditory hair cells. In 4-week-old mice, three outer hair cells (OHC) and one inner hair cell (IHC) were clearly observed (Figure 5d). In addition, even after 3 months, no damage to hair cells was observed when there was no oxidative stress (Figure 5e). The results of the histological analysis showed that there was little damage to these cells under the influence of GI (Figure 5f), moderate damage under HFD (Figure 5g), and severe damage under hypoxic conditions (Figure 5h). In addition, the analysis revealed that an OHC was close to cell death in the dual- and triple-exposure conditions (Figure 5i–k). For quantitative evaluation, we assessed the survival rate of hair cells based on the histological images from each group (Figure 5l). The survival rate was over 80% in the single condition groups and 50% in the dual condition groups, but it reduced to less than 20% when the hypoxic condition was included. In other words, oxidative stress caused by hypoxia caused damage to hair cells and led to hearing loss.

**Figure 6:**
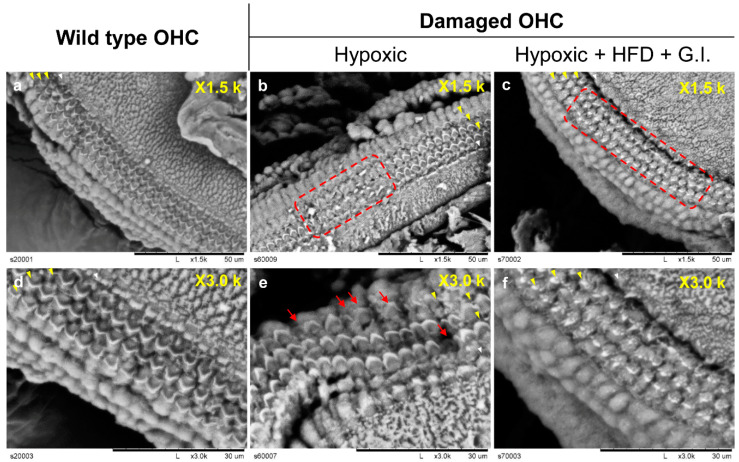
Images of OHCs and IHCs that survived in the explant model recorded by SEM. The yellow arrow indicates three OHC lines, and the white arrow indicates an IHC line. The red arrows indicate damaged hair cells. It was observed that the morphology of cilia disappeared on the line. The red dotted line indicates an extensive area of damaged hair cells. (**a–c)** microscope magnification ×1.5 k, scale bar = 50 μm; (**d–f**) microscope magnification ×3.0 k, scale bar = 30 μm. (**a**) and (**d**) show OHC images from a young mouse (4 weeks). (**b**) and (**e**) show OHCs damaged by oxidative stress due to hypoxia. (**c)** and (**f**) show OHCs damaged by oxidative stress due to hypoxia, HFD, and GI. Abbreviations: OHC, outer hair cells; IHC, inner hair cells; SEM, scanning electron microscopy; HFD, high-fat diet; GI, galactose injection.

We observed the appearance of hair cells to further examine the damage caused to them by oxidative stress. The function and survival of auditory hair cells were determined by observing the presence of stereocilia, based on previous literature [27]. Under hypoxic conditions, the cilia on the OHCs had partially disappeared (Figure 6). In addition, the damage to hair cells was severe when HFD and GI conditions were included, i.e., a triple-exposure condition. It was observed that the stereocilia disappeared almost entirely in this case, implying that auditory hair cells are damaged by oxidative stress under these conditions.

### 2.5. Expression of Age-Related Factors in Cochlea

**Figure 7:**
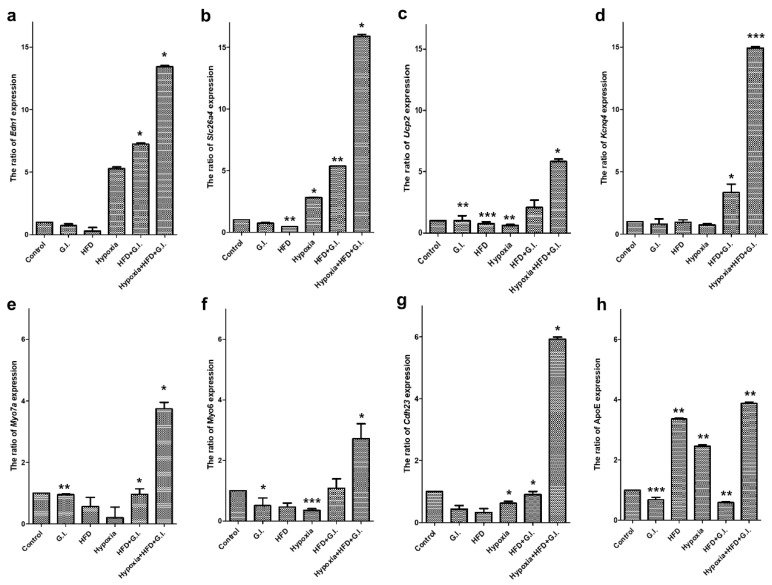
Expression of aging-related factors determined by real-time qPCR. (**a**) *Edn1*, (**b**) *Slc26a4*, (**c**) *Ucp2*, (**d**) *Kcnq4*, (**e**) *Myo7a*, (**f**) *Myo6*, (**g**) *Cdh23*, and (**h**) *ApoE*. Samples from each group show aspects of control, GI, HFD, and hypoxia (single condition), and HFD + GI, hypoxia + HFD + GI (complex conditions) from the left side. * *p* < 0.05, ** *p* < 0.01, *** *p* < 0.0001. Abbreviations: qPCR, quantitative polymerase chain reaction; HFD, high-fat diet; GI, galactose injection.

After confirming the occurrence of hearing loss due to the damage caused to hair cells by the three kinds of environmental stresses, we assessed whether factors of aging were expressed in the auditory organ. We also sought to demonstrate the age-related hearing loss caused by environmental stresses in our animal model by identifying factors that are typically expressed in ARHL. The genes *ApoE* [30,31,32] and *Edn1* [33,34] are expressed under persistent oxidative stress conditions and have been reported to be associated with vascular aging (Figure 7). In addition, *Ucp2* is the most important gene among those analyzed, and it has been reported to be expressed in mitochondrial dysfunction [18]. *Cdh23* is a gene typically expressed during ARHL [35,36]. Finally, *Kcnq4*, *Myo7a*, *Myo6*, and *Slc26a4* have been reported to be associated with the potassium channel and molecular physiological mechanisms of auditory organs [5,7,36]. All these genes are expressed during aging and are important markers that can be used to determine the cause of the expression of these genes [37].

The expression of all the selected genes increased significantly under the triple-exposure condition (Figure 7). Ion channel-related proteins in the auditory organs, such as *Slc26a4* (Figure 7b) and *Kcnq4* (Figure 7d), were overexpressed. Importantly, the expression of *Ucp2*, which is expressed during mitochondrial dysfunction due to oxidative stress, was significantly increased compared to that of other genes (Figure 7c). The expression of *Myo7a* and *Myo6* increased significantly due to the damage to the auditory organs (Figure 7e,f). Furthermore, the expression of *Cdh23*, which is the most expressed gene during the aging of auditory organs, was found to be increased as well (Figure 7g). HFD is thought to induce the expression of *ApoE* and *Edn1*, which, although not very effective, are believed to contribute to hypoxic damage (Figure 7a). In the case of *ApoE*, the results of HFD and intermittent hypoxia alone showed similar expression levels of RNA as those of exposure to all three conditions (Figure 7h). It was expected that HFD would induce hyperlipidemia in the blood vessels of the auditory organs. An imbalance in nutritional supply due to reduced blood flow has also been reported as a cause of hearing loss. Therefore, when the GI, intermittent hypoxic condition, and HFD stimulations were not performed alone, the expression of aging factors was largely observed.

## 3. Discussion

ARHL is a critical health condition that affects the aging population, and its onset varies based on the individual’s lifestyle, including eating and sleeping habits, noise exposure, and use of ototoxic drugs [4,11]. Previous studies have utilized specific genetically engineered mice or animal models of aging induced by drug injection, but these studies have been conducted without considering the changing conditions of the surrounding environment. This study describes the changes in hearing and histological phenotypes of hearing organs based on lifestyle. Herein, we show that the expression of genes associated with aging-related deafness is largely induced over a short period of time, and these genes can be explored further in preventive or therapeutic research. In other words, since it is necessary to devise an animal model suitable for such studies, we proposed an animal model that, when exposed to environmental stresses, results in hearing loss.

Hearing measurements can be obtained in an easier manner using the *C57BL/6* mice model than the other models. A recent study showed that aging is caused by oxidative stress due to changes in lifestyle, and the same study also described strategies to prevent ARHL and develop regenerative therapeutic substances [38]. This study aimed to reveal the phenotypes associated with hearing loss in a mouse model without other diseases, such as diabetes or vascular diseases. In our study, we observed that mice exposed to environmental stress for more than 3 months showed symptoms of diabetes and vascular disease; however, we did not investigate these observations further since they were beyond the scope of our study. In addition, persistent environmental stress is associated with poor quality of life, difficulty in communication, impaired activity in daily life, dementia, and cognitive dysfunction [15].

**Figure 8:**
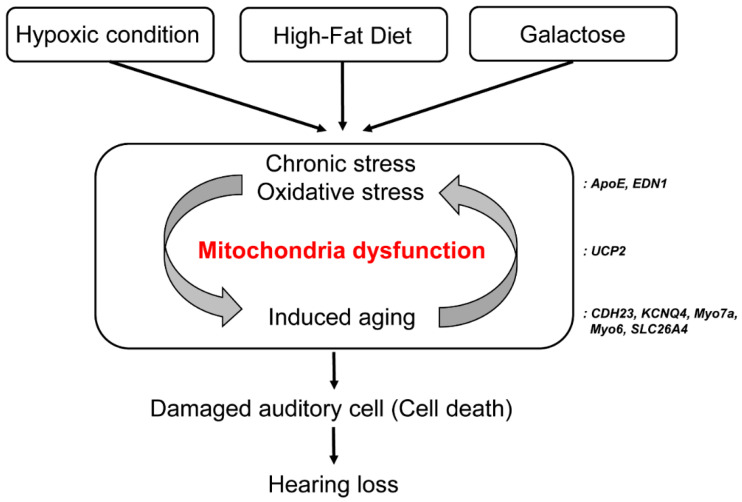
A schematic diagram of the aging mouse model exposed to three environmental stress factors. The three factors (hypoxia, HFD, and GI) cause mitochondrial dysfunction by inflicting oxidative stress on cells. Aged auditory hair cells accumulate due to irritation, followed by cell death and hearing loss. Abbreviations: HFD, high-fat diet; GI, galactose injection.

In this study, we used three kinds of environmental stress stimuli. First, a hypoxia chamber was designed based on a study of hearing loss in sleep disorders such as OSAS [16]. When oxygen saturation decreases in the body during sleep, hearing ability decreases to 60 dB or less in patients over 60 years of age [15]. We demonstrated an aged mouse model with deafness caused by the variation in confirmation of stratum corneum and wrinkles in the mouse model of oxidative stress. Changes in lifestyle that increase SOD activity in the serum and a reduction in atmospheric oxygen significantly influence aging. *Cdh23* was also increasingly expressed in the triple-exposure group (Figure 6) [39]. However, although a change in the amount of RNA was observed in *Cdh23* in this study, studies are needed to identify more accurate gene mutations. Therefore, we have suggested that environmental oxidative stress can alter the phenotype of hearing and affect certain genes. The loss of auditory hair cells and reduction in hearing ability were not significantly influenced by a single stimulus. However, when two or more stimuli were added, hearing loss was observed to occur over a short period of time. HFD significantly increases the content of fat in the body, affects sugar metabolism, restricts blood vessels, and causes metabolic diseases [19]. These effects could be demonstrated by the expression of *Edn1* and *ApoE* in cells within the hearing organ. HFD caused metabolic abnormalities in mice exposed to hypoxic conditions. Finally, 500 mg/kg galactose was injected into some mice to induce aging through metabolic abnormalities in the body, and the effects of this administration were observed in those also exposed to HFD and hypoxic conditions. By determining the expression of *Ucp2* under all environmental stimuli, it was confirmed that mitochondrial dysfunction was caused by oxidative stress [12,14]. Mitochondrial dysfunction has been observed to induce apoptosis in many studies [9,19], and we obtained similar results through histological analysis in this study. Thus, we discussed that the reduction of frequencies was influenced when the blood vessels were damaged, and substance exchange in the bloodstream was poor because of the hypoxic condition and HFD. In summary, aging and physiological changes were induced by the three lifestyle conditions considered in this study (Figure 8).

Hypoxia has the greatest effect on hearing loss induction. Meanwhile, HFD and GI induce cell nutrient supply abnormalities due to the metabolic changes they cause in the body, which promotes the aging of cells (Table 2). In this paper, it was suggested that the aging of animals due to environmental changes can be accelerated when oxidative stress is superimposed, and that hearing loss can rapidly increase after 2 months. We discussed that it is necessary to reduce oxidative stress or reduce environmental stress as candidates for therapeutic agents to slow the onset of aging hearing loss.

## 4. Materials and Methods

### 4.1. Experimental Groups

A total of 72 male mice (*C57BL/6*) were divided into eight groups based on whether they were exposed to any of the three different sources of intermittent oxidative stress or not (nonexposed mice were used as controls; Figure S3). The different groups were as follows: Group 1 (G1), normoxic, normally fed (NF); Group 2 (G2), normoxic, NF, GI; Group 3 (G3), normoxic, HFD; Group 4 (G4), normoxic, HFD, GI; Group 5 (G5), hypoxic, NF; Group 6 (G6), hypoxic, NF, GI; Group 7 (G7), hypoxic, HFD; Group 8 (G8), hypoxic, HFD, GI. Additionally, young mice (male, 4 weeks, *n* = 9) were used as controls. The body weight of the mice was monitored throughout the experiment as an indicator of health.

### 4.2. Animal Procedures

*C57BL/6* male mice (12 weeks old) were used in this study. The animal protocols used in this work were evaluated and approved by the by the institutional animal care and use committee in the animal laboratory of Yonsei University in Wonju College of Medicine (Protocol YWC-181001, Permit code: 181001-2, 2 September 2019). All animals were kept at room temperature with a 12-h light/dark cycle under different oxygen conditions, including normoxic and intermittent hypoxic conditions. They were classified according to exposure to three environmental conditions that were combinatory, leading to classification into eight groups. Groups G1 to −4 were kept under normoxic conditions with an oxygen concentration of 20%, while groups G5 to −8 were kept in a hypoxic chamber with an oxygen concentration of 5% for 12 h/day. In addition, the mice were divided into NF (NIH-41, autoclaved, Zeigler Bros Inc., Gardners, PA, USA) [40] and HFD groups [19]. The HFD groups was prepared as previously reported [18]. All materials and supporting data are listed in Table S1. Mice in groups G3, G4, G7, and G8 were fed an HFD with a fat content of 32% (Table S2), including vitamins (Table S3), to generate oxidative stress in the body, while mice of the G1, G2, G5, and G6 groups were NF (Table S4). The body metabolism changes during HFD, which promotes the aging of cells (Table S5).

In addition, mice in groups G2, G4, G6, and G8 were used to evaluate the effect of the promotion of aging through GI (500 mg/kg), which causes chronic oxidative stress and mitochondrial dysfunction. d-galactose (G0750, Sigma-Aldrich, St.Louis, MO, USA) was dissolved in a 0.9% saline solution and subcutaneously injected to induce aging in several groups (500 mg/kg) while other groups were injected with an equal volume of vehicle (0.9% saline). Body weight was measured weekly to monitor changes. Furthermore, the threshold of hearing was evaluated by measuring ABR every 2 weeks. To check the oxidative stress in the body, serum samples were collected every month.

### 4.3. Hypoxia Chamber Design

The hypoxia chamber was designed to inflict chronic oxidative stress in the mice (Figure S4). This chamber was made of acrylic sheets and consisted of a fan that automatically injected fresh air and nitrogen into the upper part of the chamber and dispersed the air (Figure S5). The chamber (340 × 240 × 60 mm, internal volume 4.9 L) was designed to control oxygen concentration via nitrogen injection with a LCI system (Live Cell Instrument Co., Seoul, Korea) in accordance with previous literature [16,41]. This chamber automatically maintained the oxygen concentration at either about 20% or 5% using nitrogen at a 12-h time split. Nitrogen was automatically injected every 2 min during the 12 h period to decrease the oxygen concentration to 5%. After 12 h, fresh air was injected to allow sufficient oxygen supply for 12 h. In total, a 24-h reaction was performed in each cycle, and the concentration of oxygen was recorded every second per day (Figure S4b,c). All the used gas was discharged out of the building via tubes, and an oxygen indicator was used to check the amount of oxygen in the hypoxia chamber. Inside the chamber, walls were created to divide the NF and HFD groups to distinguish the type of feeding (Figure S4d). The cages were cleaned once a week, and the food, water, and mice feces were removed.

### 4.4. Auditory Brainstem Response

All mice were anesthetized with 100 mg/kg ketamine (Yuhan-Kimberly, Seoul, Korea) and 10 mg/kg xylazine hydrochloride (Rompun, Bayer, Ansan, Korea) by intraperitoneal injection before ABR recording. Auditory brainstem responses (ABR) are auditory evoked potentials derived from the activity of the auditory nerve and the central auditory pathways in response to transient sound (auditory clicks or tone pips). The thresholds of the ABR wave V were determined by progressively attenuating the sound intensity in 5 dB steps from 80 dB SPL until wave V was no longer distinguishable from the noise floor in recorded traces.

Mice were tested in a sound-attenuating chamber with a built-in Faraday cage, and an isothermal pad was used to maintain the body temperature. The TDT RZ6/BioSigRZ system (Tucker Davis Technologies, Alachua, FL, USA) was used for stimulus generation, data management, and ABR collection [42]. Subdermal electrodes were placed in each mouse for data collection. The reference electrode, which was on the same side as the stimulus, was placed axial to the pinnae, while the ground electrode was placed in the ipsilateral ear. Meanwhile, the active electrodes were placed at the vertex. The ABR test was conducted every 2 weeks to assess the stability of the experimental group.

### 4.5. Superoxide Dismutase (SOD) Activity Test

SOD was rapidly measured using blood collected from the retro-orbital plexus of mice in each group. The collected blood was allowed to clot in an anticoagulant tube for 30 min at 25 °C (room temperature, RT). Purified serum was obtained after the blood was centrifuged at 2000× *g* for 15 min at 4 °C. All samples were stored at −80 °C for a month. Working samples were diluted in the ratio of 1:5 with sample buffer before assaying for SOD activity. The SOD Assay kit (No. 706002, Cayman Che., MI, USA) uses a tetrazolium salt for the detection of superoxide radicals generated by xanthine oxidase and hypoxanthine [43]. For each sample, SOD activity was calculated using the equation obtained from the linear regression of the standard curve, replacing the linearized rate. One unit was defined as the amount of enzyme required to represent 50% displacement of the superoxide radical. SOD activity was measured according to the manufacturer’s method.

### 4.6. Histological Analysis

All mice were sacrificed by cervical dislocation, and both cochleae were dissected. The cochleae were perfused with a fixative containing 4% paraformaldehyde in phosphate-buffered saline (PBS; pH 7.4) at RT. The apical portion of the bony cochlea was gently opened to allow the fixative to perfuse through the tissues. The cochleae were decalcified by immersion in a Calci-Clear rapid decalcifier (National Diagnostics, Atlanta, GA, USA) for 24 h. Thereafter, the cochleae were embedded in a compound at optimal cutting temperature (Leica Microsystems, Bensheim, Germany), and cut into 2- to 5-μm-thick sections in a LEICA RM2145 (Leica Biosystems, Wetzlar, German). The cut sections were subjected to standard hematoxylin and eosin (H&E) staining (1–3 min of incubation in hematoxylin, and staining with eosin for 30–60 s).

### 4.7. Immunostaining

The cochlear sections were prepared on 5-µm-thick gelatine-coated slides by fixing them with 4% paraformaldehyde for 15 min and allowing them to dry at RT. The specimens were incubated in 5% normal goat serum for 1 h at RT to prevent nonspecific labeling. Then, the specimens were incubated with the primary antibody, MYO7A (1:200, ab3481, Abcam, UK), for 1 h at 4 °C [44,45,46]. Thereafter, the specimens were washed with PBS three times for five min each time, followed by incubation with a secondary antibody, goat anti-rabbit IgG H&L (Alexa Fluor^®^ 488; 1:1000, ab150077, Abcam, UK), for 1 h at RT. After washing the samples three times for five min with PBS again, they were finally immobilized with a mounting solution containing DAPI (4′,6-diamidino-2-phenylindole). All the samples were observed by confocal microscopy (Carl Zeiss Microscopy GmbH, Jena, Germany), and images were analyzed using the software ZEN lite ver. 2.3 (ZEN lite, Jena, Germany).

### 4.8. Scanning Electron Microscope (SEM)

The cochlea was isolated to observe the morphology of the hair cells. The cochlea was extracted from the auditory organ and fixed in 2.5% glutaraldehyde for 2 h at 4 °C. The specimens were then fixed in 1% osmium tetroxide (OsO_4_) after being washed twice with 0.1 M cacodylate buffer. The dehydration steps were performed using 50%, 70%, 80%, 90%, and 100% ethanol. Then, the samples were set to react with 3-methylbutyl acetate (Isoamyl acetate, Hanawa, Japan) for 15 min at RT. The samples were dried using hexamethyldisilazane (cat. 440191, Sigma-Aldrich, USA) for 15 min at RT. All samples were air-dried overnight on covered filter papers with a lid. The samples were gold-coated to observe the morphology of the cochlea using a tabletop microscope (TM-1000, Hitachi Ltd., Tokyo, Japan) [46].

### 4.9. Real-Time PCR

Real-time PCR was performed to determine the induction of aging and hearing loss by analyzing the expression of various genes in the liver, kidney, and cochlea. Total RNA was extracted using TRIzol (Thermo Fisher Scientific, San Diego, CA, USA). To prepare the mRNA samples, 2 µL of mRNA and 8 µL of reverse transcriptase reagents, which comprised 1 µL of 10× enzyme mix, 2 µL of 5× enzyme reaction buffer, and 5 µL of nuclease-free water, were used to prepare a 10-µL mixture according to the manufacturer’s protocol. cDNA was diluted in a ratio of 1:10 with 90 µL nuclease-free water for microRNA real-time PCR. RT-PCR was performed using the Applied Biosystems 7900 HT sequence detection system (Thermo Fisher Sci. San Diego, CA, USA). Samples were subjected to reverse transcription using the SYBR Select master mix (Applied Biosystems, Calrsbad, CA, USA), following the manufacturer’s protocol. The sequences of the primers used were as follows (Table S6): apo-lipoprotein E (*ApoE*), forward: 5′-GGT TCG AGC CAA TAG TGG AA–3′, and reverse: 5′-ATG GAT GTT GCA GGA CA-3′; Cadherin-23 (*Cdh23*), forward: 5′-ATG GAG AGC CCT CTG GAA AT-3′, and reverse: 5′-ACC CAC AAA GGC TGT ACT GG-3′; eosinophil-derived neurotoxin 1 (*Edn1*), forward: 5′-ACA CCG TCC TCT TCG TTT TG-3′, and reverse: 5′-GAG TC CTT GGA AAG TCA CG-3′; potassium voltage-gated channel subfamily Q member 4 (*Kcnq4*), forward: 5′-TGT TGG GAT CCG TGG TCT AT -3′, and reverse: 5′- GAGTTG GCA TCC TTC TCA GC-3′; myosin VIIA (*Myo7a*), forward: 5′-GAC AAC TCT AGC CGC TTT GG-3′, and reverse: 5′-GAC ACG TGA CTT CTC CAG CA-3′; myosin VI (*Myo6*), forward: 5′-AGA CCA CTT CCG GCT CAC TA-3′, and reverse: 5′- TGG GTT GTC TCG TAG CAC AC-3′; uncoupling protein 2 (*Ucp2*), forward: 5′-CTC AAA GCA GCC TCC AGA AC-3′, and reverse: 5′-ACA TCT GTG GCC TTG AAA CC-3′; solute carrier family 26 member 4 (*Slc26a4*), forward: 5′-TCA TTG CCT TTG GGA TAA GC-3′, and reverse: 5′-GGC AAC CAT CAC AAT CAC AG-3′; *18S* rRNA, forward: 5′-CAT TCG AAC GTC TGC CCT AT-3′, and reverse: 5′-GTT TCT CAG GCT CCC TCT CC-3′. The total 10 µL sample for real-time PCR contained 1 µL cDNA, 5 µL of premix, 1 µL each of 10 pmol forward and reverse primers, and 3 µL of nuclease-free water. The PCR conditions comprised preamplification at 95 °C for three min, 40 cycles of 95 °C for 10 s, and then 60 °C for 60 s, and melting curve analysis. All processes were performed in duplicate. The normalization of mRNA expression level was calculated using *18S* rRNA.

### 4.10. Statistical Analysis

Statistical analysis was performed using SPSS statistical package version 21.0 (SPSS Inc. Chicago, IL, USA). Descriptive results of continuous variables were expressed as the mean ± standard deviation for normally distributed variables. Mean hearing thresholds were compared by two-way ANOVA. In addition, the sensitivity and specificity of the statistically significant mRNAs were analyzed by the Mann–Whitney U-test with GraphPad PRISM version 5.0 (GraphPad Inc., La Jolla, CA, USA). *p*-values less than 0.05 were considered to be statistically significant.

## 5. Conclusions

Interestingly, exposure to HFD or GI as a single condition showed no significant hearing loss. However, when hypoxic stimulation was added, the dual condition showed intermediate effects, and the triple-exposure condition showed the maximum effects. In addition, OHC loss for high sound frequencies occurs when the blood vessels are damaged and the exchange of substances in the bloodstream is poor. This can cause ion channel abnormalities and genetic defects in the auditory organs. With the new model developed in this study, which causes natural short-term induction of ARHL due to oxidative stress through changes in the lifestyle, we could observe the age-related markers and phenotypes associated with ARHL. The animal model used and results reported in this study can aid in the development of strategies for the prevention and treatment of ARHL. With the increase in the aged population, advances in medical technologies and research on hearing loss are warranted.

## Figures and Tables

**Table 1:** Monthly measurement of SOD activity in serum.

Groups	0 Month	1 Month	2 Months	3 Months
1	0.6325 ± 0.000	0.5873 ± 0.005	0.5667 ± 0.000	0.5311 ± 0.019
2	0.5955 ± 0.017	0.5463 ± 0.005	0.5216 ± 0.005	0.5148 ± 0.028
3	0.5586 ± 0.011	0.5463 ± 0.005	0.5298 ± 0.005	0.4901 ± 0.011
4	0.5873 ± 0.005	0.5298 ± 0.005	0.5011 ± 0.011	0.5572 ± 0.050
5	0.5873 ± 0.029	0.5463 ± 0.005	0.5463 ± 0.005	0.6215 ± 0.048
6	0.5914 ± 0.000	0.5627 ± 0.005	0.5463 ± 0.005	0.5695 ± 0.059
7	0.5996 ± 0.000	0.5463 ± 0.005	0.5914 ± 0.000	0.6435 ± 0.055
8	0.5750 ± 0.011	0.5463 ± 0.005	0.5380 ± 0.005	0.5955 ± 0.022

Abbreviation: SOD, superoxide dismutase.

**Table 2:** Phenotypic changes caused by exposure to three environmental stresses (hypoxia, HFD, GI).

	Merged Condition			Effect of a Single Factor		
	Triple	Dual	Single	Hypoxia	HFD	GI
Phenotype						
Body weight	+++	++	+	++	+++	+
Skin thickness	+++	++	+	++	+++	+
Hair cell loss	+++	++	+	+++	++	+
Oxidative stress	+++	++	+	+++	++	+
Hearing loss	+++	++	+	+++	++	+
Age-related gene expression	+++	++	+	+++	++	+

Abbreviations: HFD, high-fat diet; GI, galactose injection.

## Supplementary Materials

The following are available online at https://www.mdpi.com/1422-0067/21/19/7068/s1, Figure S1: Phenotype of mice groups, Figure S2: Two-way ANOVA of hearing threshold at three frequencies (8, 16, and 24 kHz) depending on the duration, respectively. Figure S3: Overall design, including the mouse housing conditions and ABR test. Figure S4: Hypoxia chamber design for oxidative stress., Figure S5: Overall appearance of the hypoxic chamber. Table S1: Thickness of the dermis and fat layers between different groups. Table S2: Ingredient Composition of High-Fat Diet 32 (HFD32). Table S3: Ingredient composition of AIN93-VX vitamin mix and AIN93G mineral mix. Table S4: Ingredients and nutrient composition in experimental chows (NIH-41). Table S5: Compared guaranteed analysis between NIH41 and HFD32 (%). Table S6: Primers of target genes for aging.

## Abbreviations

ABR	Auditory brainstem response
ARHL	Age-related hearing loss
GI	Galactose injection
H&E	Haematoxylin and eosin
HFD	High-fat diet
IHC	Inner hair cell
KHIDI	Korea Health Industry Development Institute
NF	Normally fed
OC	Organ of Corti
OHC	Outer hair cell
OSAS	Obstructive sleep apnoea syndrome
ROS	Reactive oxidative stress
RT	Room temperature
