# Supplementary material for: Induced Short-Term Hearing Loss due to Stimulation of Age-Related Factors by Intermittent Hypoxia, High-Fat Diet, and Galactose Injection
Source: Int J Mol Sci. 2020 Sep 25;21(19):7068. doi: 10.3390/ijms21197068 (PMC7582260; doi:10.3390/ijms21197068)
Supplement: Supplementary file 1 [file ijms-21-07068-s001.pdf]

Induced Short-Term Hearing Loss due to Stimulation of Age-Related Factors by Intermittent Hypoxia, High-Fat Diet, and Galactose Injection

Dong Jun Park [1], Sunmok Ha [2], Jin Sil Choi [1], Su Hoon Lee[1], Jeong-Eun Park [3] and Young Joon Seo [1,*]

[1] Department of Otorhinolaryngology, Yonsei University Wonju College of Medicine, 20 Ilsan-ro, Wonju, Gangwon-do 26426, South Korea.

[2] Department of Biomedical Laboratory Science, College of Health Sciences, Yonsei University, wonju, Republic of Korea.

[3] Department of Otorhinolaryngology Head and Neck Surgery, Hallym University College of Medicine, Dongtan Sacred Heart Hospital, Hwaseong, South Korea.

* Correspondence: okas2000@hanmail.net; Tel.: +82-33-741-0644 (Y.J.S.)

(a) Normoxic vs. hypoxic (Gal (-) vs Gal(+))

	Normoxic	Hypoxic			
Feed	NF	NF		HFD	
G.I.	(-)	(-)	(+)	(-)	(+)
Group	G1	G5	G6	G7	G8

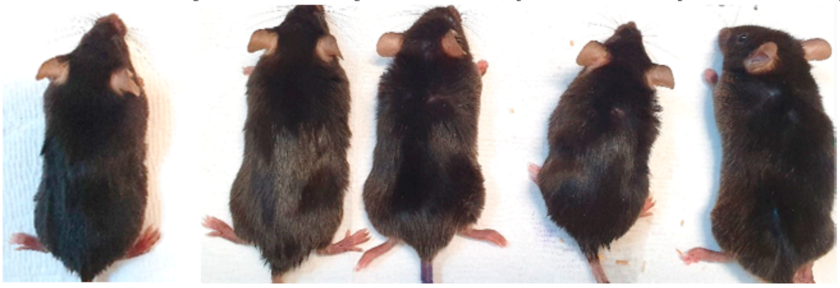

(b) NF vs. HFD

Normoxic		Hypoxic	
NF	HFD	NF	HFD
G1	G3	G5	G7

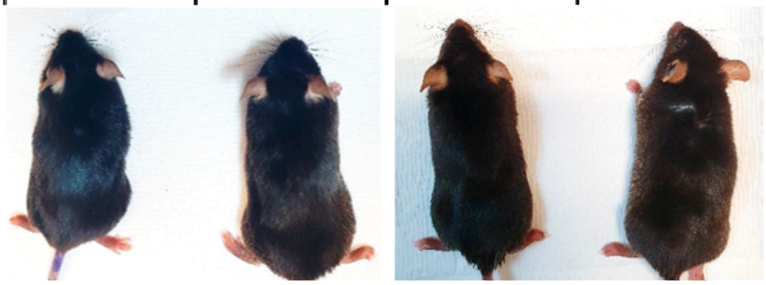

Figure S1. Phenotype of mice groups (HFD, GI, Hypoxic). (**a**) Appearance of mice of the groups based on the three conditions. (**b**) Appearance of mice of the groups based on NF vs HFD plus normoxia vs hypoxia.

Abbreviations: HFD, high-fat diet; GI, galactose injection; NF, normally feed.

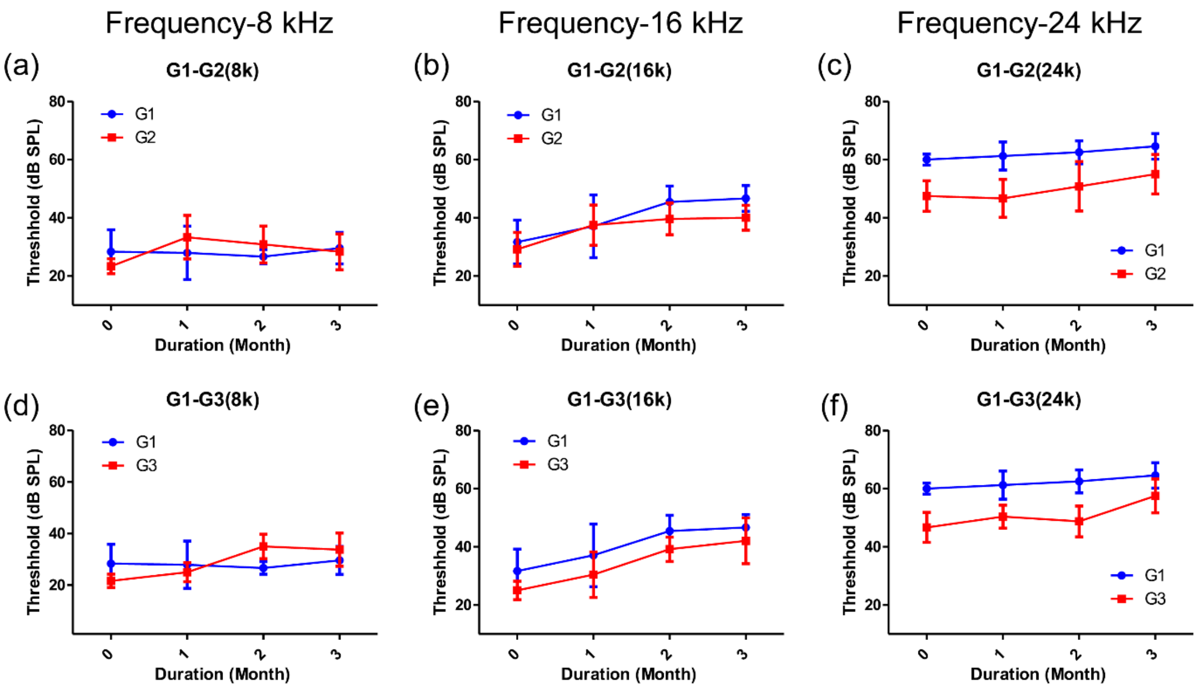

Figure S2. Two-way ANOVA of hearing threshold at three frequencies (8, 16, and 24 kHz) depending on the duration, respectively. (**a**) The average of hearing thresholds represents G1 and G2 at 8 kHz. (**b**) The average of hearing thresholds represents G1 and G2 at 16 kHz. (**c**) The average of hearing thresholds represents G1 and G2 at 24 kHz. (**d**) The average of hearing thresholds represents G1 and G3 at 8 kHz. (**e**) The average of hearing thresholds represents G1 and G3 at 16 kHz. (**f**) The average of hearing thresholds represents G1 and G3 at 24 kHz.

Abbreviation: ANOVA, analysis of variance.

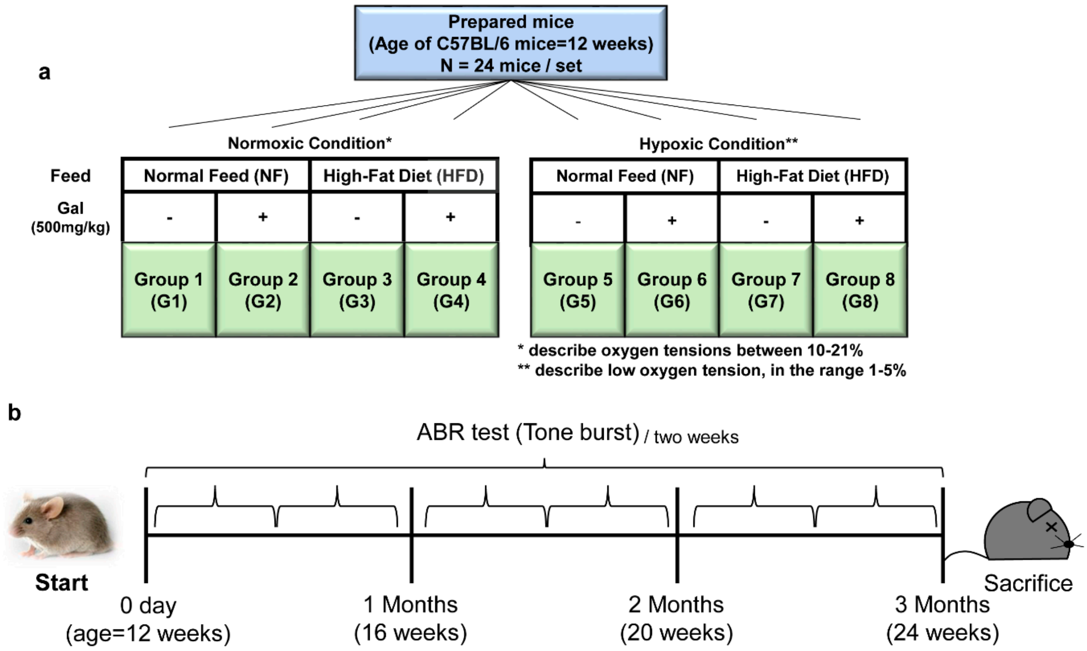

Abbreviations: ABR, auditory brainstem response; HFD, high-fat diet; GI, galactose injection; NF, normally fed.

Figure S3. Overall design, including the mouse housing conditions and ABR test. (**a**) Classification of groups based on hypoxic condition (≤ 5%), HFD, and GI (500 mg/kg). Mice (n = 72) were divided into eight groups in this study. G1 (Normoxic, NF), G2 (Normoxic, NF, GI), G3 (Normoxic, HFD), G4 (Normoxic, HFD, GI), G5 (Hypoxic, NF), G6 (Hypoxic, NF, GI), G7 (Hypoxic, HFD), and G8 (Hypoxic, HFD, GI). (**b**) The ABR test for all mice was performed once every 2 weeks. Starting at 12 weeks of age, all mice were sacrificed by cervical dislocation after 3 months from when hearing loss was measured.

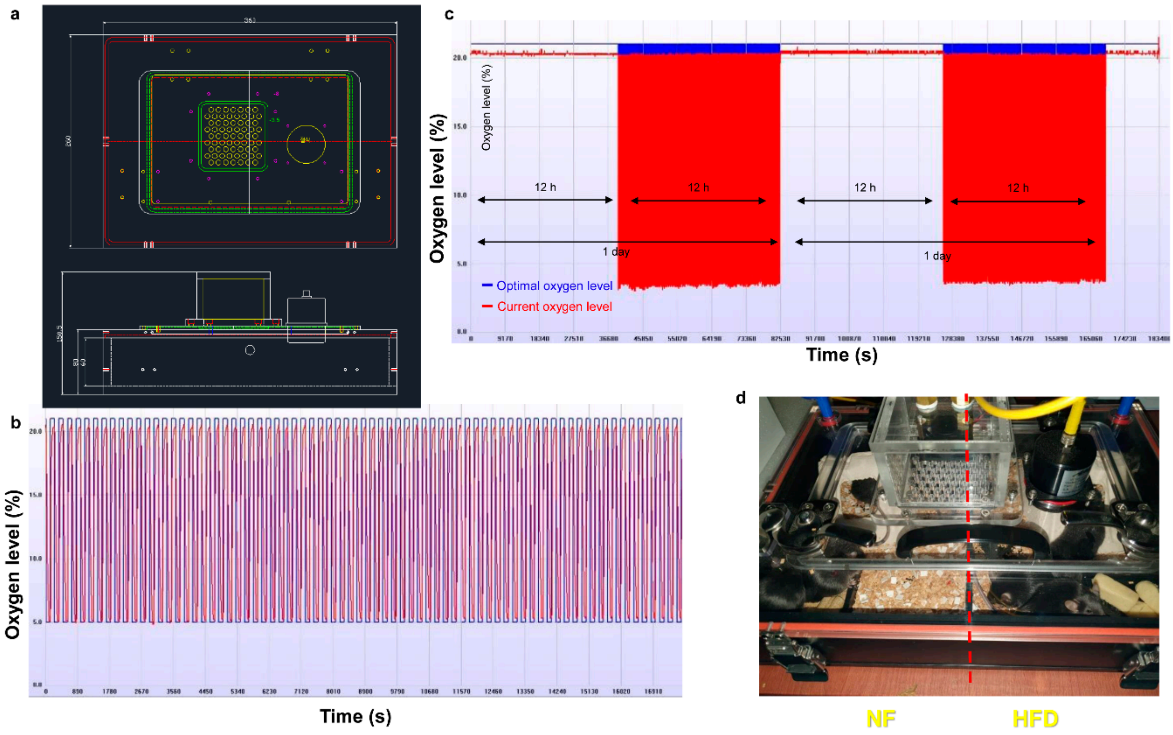

Abbreviations: HFD, high-fat diet; NF, normally fed.

Figure S4. Hypoxia chamber design for oxidative stress. (**a**) Blueprint of the hypoxia chamber for mice. (**b**) Measurement of automatic change in oxygen levels in the hypoxia chamber from 20% to 5%. The blue line represents the amount of oxygen set by the machine, and the red line represents the actual amount of oxygen. Nitrogen was automatically injected into the chamber to alter the oxygen levels. (**c**) Daily analysis of the oxygen level in the chamber. Oxygen was cycled through the chamber to achieve the hypoxic condition (≤ 5%) for 12 h a day and the normoxic condition (20%) for 12 h a day. (**d**) The mice were physically divided by the middle wall in the hypoxia chamber. Mice on the left side of the chamber were NF, while those on the right side were fed HFD.

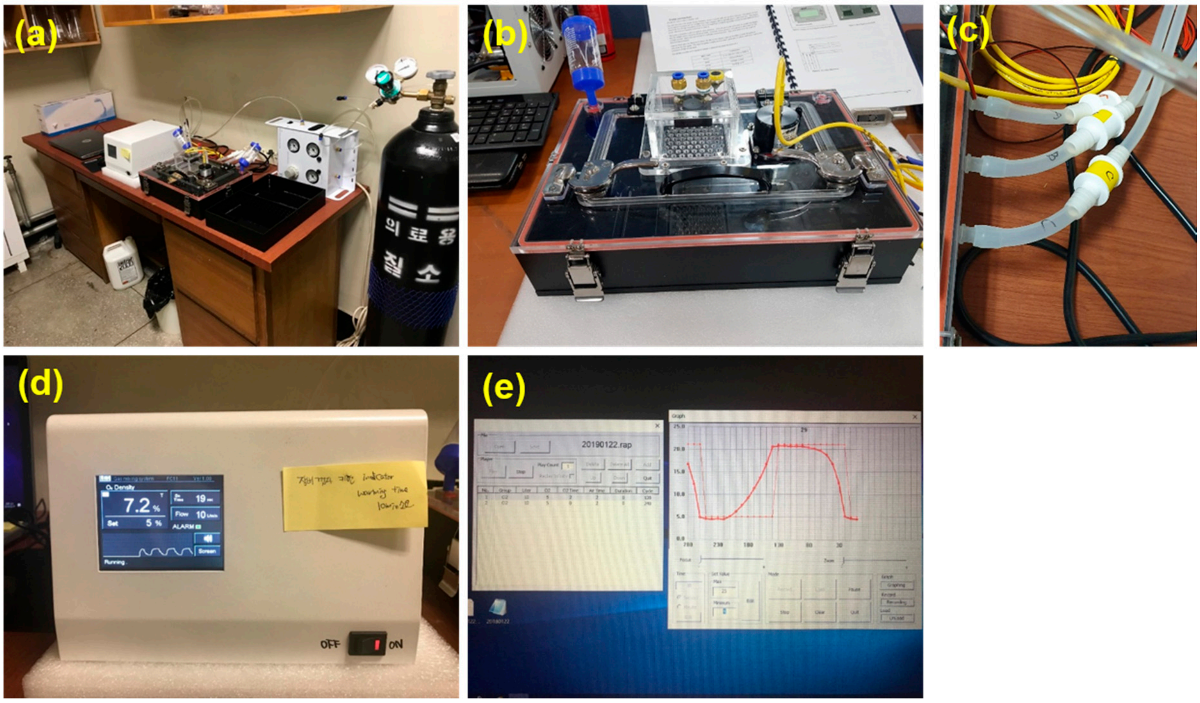

Figure S5. Overall appearance of the hypoxic chamber. (**a**) Installation of hypoxic chamber with nitrogen tank, (**b**) Appearance of hypoxic chamber, (**c**) Nitrogen injection and discharge nozzle, (**d**) Oxygen level meter in the hypoxic chamber, (**e**) Measurement of oxygen change in the hypoxic chamber.

Table S1. Thickness of the dermis and fat layers between different groups.

	WT	+GI	+HFD	+HFD, GI	+Hypoxic	+Hypoxic, HFD, GI
**Dermis Thickness**	287.05±99.26	348.5±85.9	346.76±81.53	392.35±67.59	351.4±93.46	388.42±66.9
**Fat layer Thickness**	87.59±19.76	127.4±29.04	600.29±18.18	594.44±18.55	89.71±36.00	133.67±36.87

Abbreviations: WT, wild-type; HFD, high-fat diet;

Table S2. Ingredient Composition of High-Fat Diet 32 (HFD32).

Ingredient Composition	% per weight
Milk casein	24.5
Egg white	5
L-cystine	0.43
Powdered beef tallow (including 80% of beef tallow)	15.88
Safflower oil (high oleic acid)	20
Crystalline Cellulose	5.5
Maltodextrin	8.25
Lactose	6.928
Sucrose	6.75
AIN93 vitamin mix	1.4
AIN93G mineral mix	5
Choline bitartrate	0.36
Teriary butylhydroquinone	0.002
Total	100

Abbreviation: HFD, high-fat diet.

Table S3. Ingredient composition of AIN93-VX vitamin mix and AIN93G mineral mix.

a. Ingredient composition of AIN93-VX vitamin mix

Ingredient Composition	*g per weight*
Niacin	3.0
Calcium Pantothenate	1.6
Pyridoxine HCl	0.7
Thiamin (81%)	0.6
Riboflavin	0.6
Folic Acid	0.2
Biotin	0.02
Vitamin B12(0.1% in mannitol)	2.5
Vitamin E, DL-alpha tocopheryl acetate (500IU/g)	15.0
Vitamin A Palmitate (500,000IU/g)	0.8
Vitamin D3, Cholecalciferol (500,000 IU/g)	0.2
Vitamin K1, Phylloquinone	0.075
Sucrose, fine ground	974.705
Total	1000

b. Ingredient composition of AIN93G mineral mix

Ingredient Composition	*g per weight*
Calcium Carbonate	357.0
Potassium Phosphate	196.0
Potassium Citrate	70.78
Sodium Chloride	74.0
Potassium Sulfate	46.6
Magnesium Oxide	24.3
Ferric Citrate	6.06
Zinc Carbonate	1.65
Magnanous Carbonate	0.63
Cupric Carbonate	0.31
Potassium Lodate	0.01
Sodium Selenate	0.0103
Ammonium Paramolybdate	0.008
Sodium Meta-Silicate	1.45
Chromium Potassium Sulfate	0.275
Lithium Chloride	0.0174
Boric acid	0.0815
Sodium Fluoride	0.0635
Nickel Carbonate Hydroxide	0.0318
Ammonium Meta-vanadate	0.0066
Sucrose	220.7159
Total	1000

Table S4. Ingredients and nutrient composition in experimental chows (NIH-41).

Ingredient (Unit)	*% per weight*
Ground whole wheat	34.9
Ground No. 2 Yellow Corn	21.0
Ground whole oats	10.0
Wheat Middlings	10.0
Fish Meal (60% protein)	9.0
Soybean Meal (45% protein)	5.0
Soy Oil	2.0
Alfalfa Meal (17% protein)	2.0
Corn gluten meal (60% protein)	2.0
Dicalcium phosphate	1.5
Brewers dried east	1.0
Premixes	0.6
Limestone	0.5
Salt	0.5
Amino Acid Concentration	% of total diet
Arginine	0.9
Lysine	0.85
Methionine	0.35
Cystine	0.25
Tryptophan	0.2
Glycine	0.95
Histidine	0.38
Leucin	1.4
Isoleucine	0.95
Phenylalanine	0.85
Tyrosine	0.6
Threonine	0.65
Valine	0.9
Glycine	0.95
Mineral Concentration (Unit)	per weight
Calcium (%)	1
Phosphorous (%)	0.94
Potassium (%)	0.55
Sodium (%)	0.25
Magnesium (%)	0.15
Iron (ppm)	300
Zinc (ppm)	40

Manganese (ppm)	140
Copper (ppm)	12
Cobalt (ppm)	0.7
Iodine (ppm)	1.8
Vitamin Concentration (Unit)	per weight
Vitamin A (IU/g)	17
Vitamin D3 (IU/g)	4
Alpha-Tocopherol (IU/g)	45
Thiamine (ppm)	15
Riboflavin (ppm)	9
Niacin (ppm)	70
Pantothenic Acid (ppm)	30
Choline (ppm)	1900
Folic Acid (ppm)	2
Biotin (ppm)	2
Vitamin B12 (Mcg/kg)	75
Vitamin K (ppm)	2

Table S5. Compared guaranteed analysis between NIH41 and HFD32 (%).

Guaranteed Analysis	NIH-41	HFD32
Moisture with vitamins	36.9	6.2
Crude Protein	18.0	25.5
Crude Fat	5.0	32.0
Crude Fiber	5.0	2.9
Ash (%)	35.1	4.0
NFE (%)	-	29.4
Total	100	100

Abbreviation: NFE, nitrogen-free extract

Table S6. Primers of target genes for aging.

Target gene	Predicable Dysfunctional effect	F/R	5’ → 3’
ApoE	Age-related disorders, Vascular disease	F	GGT TCG AGC CAA TAG TGG AA
		R	ATG GAT GTT GTT GCA GGA CA
Edn1	Cardiovascular complications	F	ACA CCG TCC TCT TCG TTT TG
		R	GAG TC CTT GGA AAG TCA CG
Ucp2	Mitochondria dysfunction	F	CTC AAA GCA GCC TCC AGA AC
		R	ACA TCT GTG GCC TTG AAA CC
Cdh23	Age-related hearing loss	F	ATG GAG AGC CCT CTG GAA AT
		R	ACC CAC AAA GGC TGT ACT GG
Kcnq4	Non-syndromic sensorineural hearing loss	F	TGT TGG GAT CCG TGG TCT AT
		R	GAGTTG GCA TCC TTC TCA GC
Myo7a	Non-syndromic hearing loss and deafness	F	GAC AAC TCT AGC CGC TTT GG
		R	GAC ACG TGA CTT CTC CAG CA
Myo6	Non-syndromic hearing loss and deafness	F	AGA CCA CTT CCG GCT CAC TA
		R	TGG GTT GTC TCG TAG CAC AC
Slc26a4	Anion transport dysfunction in auditory organ	F	TCA TTG CCT TTG GGA TAA GC
		R	GGC AAC CAT CAC AAT CAC AG
18s	Ribosomal RNA	F	CAT TCG AAC GTC TGC CCT AT
		R	GTT TCT CAG GCT CCC TCT CC
